# Bilobalide Exerts Anti-Inflammatory Effects on Chondrocytes Through the AMPK/SIRT1/mTOR Pathway to Attenuate ACLT-Induced Post-Traumatic Osteoarthritis in Rats

**DOI:** 10.3389/fphar.2022.783506

**Published:** 2022-02-23

**Authors:** Tianwen Ma, Liangyu Lv, Yue Yu, Lina Jia, Xiaopeng Song, XinYu Xu, Ting Li, Xuanbo Sheng, Haoran Wang, Jiantao Zhang, Li Gao

**Affiliations:** ^1^ College of Veterinary Medicine, Northeast Agricultural University, Harbin, China; ^2^ Heilongjiang Key Laboratory of Animals Disease Pathogenesis and Comparative Medicine, Harbin, China

**Keywords:** bilobalide, autophagy, AMPK/SIRT1/mTOR, inflammation, osteoarthritis, ACLT

## Abstract

Although osteoarthritis (OA) significantly affects the quality of life of the elderly, there is still no effective treatment strategy. The standardized *Ginkgo biloba* L. extract preparation has been shown to have a wide range of therapeutic effects. Bilobalide, a unique ingredient of *Ginkgo biloba*, has anti-inflammatory and antioxidant pharmacological properties, but its mechanism of action on OA remains unknown. In this study, we investigated the effects of bilobalide on the development of OA through *in vivo* and *in vitro* experiments, as well as its potential anti-inflammatory mechanisms. The *in vitro* experiments demonstrated that bilobalide significantly inhibited the production of inducible nitric oxide synthase (iNOS), cyclooxygenase-2 (COX-2), and matrix metalloproteinase 13 (MMP13) in ATDC5 chondrocytes induced by Interleukin-1β (IL-1β). At the molecular level, bilobalide induced chondrocyte autophagy by activating the AMPK/SIRT1/mTOR signaling pathway, which increased the expression of autophagy-related Atg genes, up-regulated the expression of LC3 protein, and reduced the expression of the p62 protein. *In vivo*, bilobalide exerted significant anti-inflammatory and anti-extracellular matrix (ECM) degradation effects in a rat model of post-traumatic OA (PTOA) induced by anterior cruciate ligament transection (ACLT). Bilobalide could relieve joint pain in PTOA rats, inhibit the expression of iNOS and COX-2 protein in cartilage via the AMPK/SIRT1/mTOR pathway, and reduce the level of ECM degradation biomarkers in serum. In conclusion, bilobalide exhibits vigorous anti-inflammatory activity, presenting it as an interesting potential therapeutic agent for OA.

## 1 Introduction

Osteoarthritis (OA) is an age-related, painful, and disabling disease. Around 35% of the global population over the age of 60 suffers from symptomatic (painful, disabling) OA (Grassel et al., 2021). OA may develop in any joint, such as the shoulders, hands, feet, spine, knees, and hip joints (Katz et al., 2021). Currently, Non-steroidal anti-inflammatory drugs (NSAIDs) and tramadol are recommended for the clinical treatment of OA. However, NSAIDs have gastrointestinal and cardiovascular side effects, are commonly used in clinical practice for the short-term treatment of OA (Chen et al., 2008). Safe and effective drugs for preventing or reversing the progression of OA are therefore still lacking (Pérez-Lozano et al., 2021).

Notably, plant extracts have attracted a lot of attention due to their safe and diverse biological activities (Sukhikh et al., 2021) (Henrotin and Mobasheri, 2018). *Ginkgo biloba* L. extract is one of the most widely used botanical drugs in the world (Achete de Souza et al., 2020). Various biological preparations made from *Ginkgo biloba* L. extract as raw materials are commonly used in medicines, dietary supplements, food additives, cosmetics, and functional beverages (Mahadevan and Park, 2008; dal Belo et al., 2009; Aziz et al., 2018; Palacios et al., 2019). Bilobalide is a sesquiterpene compound extracted from *Ginkgo biloba* L. that has received widespread interest due to its anti-inflammatory (Jiang et al., 2014), anti-oxidative (Achete de Souza et al., 2020), and neuroprotective properties (Feng et al., 2019). Bilobalide has been shown to inhibit the production of pro-inflammatory mediators, improve mitochondrial function, and prevent damage caused by cerebral ischemia (Jiang et al., 2014). Recent studies indicate that bilobalide inhibits IL-17-induced inflammatory damage in ATDC5 cells by down-regulating microRNA-125a *via* the JNK and NF-kB signaling pathways (Mao et al., 2019), demonstrating that bilobalide has anti-inflammatory effects on chondrocytes. However, further studies on its molecular mechanism are required.

Autophagy is a widely occurring cell self-protection mechanism that acts as an intracellular scavenger to maintain intracellular balance in eukaryotic cells (Kang and Elledge, 2016; Vinatier et al., 2018). The activation of autophagy has positive significance for the survival of chondrocytes in the early stage of OA (Duan et al., 2020). During the development of OA, decreased chondrocyte autophagy leads to impaired cellular function, resulting in joint aging and dysfunction (Netea-Maier et al., 2016), indicating that autophagy plays an important role in the development of OA and is expected to be an important target for OA treatment. Several studies have reported that bilobalide can interfere with certain diseases by regulating autophagy. Bilobalide inhibits LPS-induced neuroinflammation and promotes autophagy (Qin et al., 2021). After cerebral ischemia/reperfusion injury in rats, treatment with bilobalide can significantly reduce cell apoptosis and autophagy, while promoting angiogenesis (Zheng et al., 2018). These findings indicated that the dynamic process of regulating autophagy and anti-inflammatory can delay the development of certain diseases, although it remains unclear whether bilobalide inhibits the inflammatory response of OA via the autophagy pathway.

Adenosine Monophosphate Activated Protein Kinas (AMPK) is an energy sensor that monitors and responds to changes of AMP/ATP ratio in the intracellular (Asby et al., 2015). Sirtuin 1 (SIRT1), a downstream signaling molecule of AMPK, has been shown to trigger the formation of autophagosomes by inducing the expression of autophagy-related proteins (Lee et al., 2008; Mengqi Zhang et al., 2020). The activity of SIRT1 can be positively regulated by AMPK (Wang et al., 2020). Recent studies have found that abnormal AMPK/SIRT1 activity is linked to the development of OA (Mei et al., 2020; Jiang et al., 2021; Zheng et al., 2021). It has been demonstrated that AMPK serves as a potent activator of autophagy to protect chondrocytes from cellular stress. In the nutrient-poor context, active AMPK phosphorylates, an essential component for mTOR activation, and then directly inhibits mTOR, which induces autophagy (Holczer et al., 2019). Moreover, the AMPK/SIRT1/mTOR pathway is known to be associated with autophagy and may be a potential pharmacological target for OA.

In this study, we focused on 1) the anti-inflammatory and anti-ECM degradation effects of bilobalide in IL-1β-induced ADTC5 chondrocytes, and 2) its protective effects in an anterior cruciate ligament transection (ACLT)-induced rat model of post-traumatic OA (PTOA) and its underlying mechanism. This study aimed to provide new insights for the treatment of OA and lay the foundation for future clinical applications of bilobalide.

## 2 Materials and Methods

### 2.1 Cell Culture and Treatment

ATDC5 cells were purchased from Shanghai Zhong Qiao Xin Zhou Biotechnology Co., Ltd. (Shanghai, China). The cells were cultured in Dulbecco’s Modified Eagle Medium/Nutrient Mixture F-12 (DMEM/F12) medium (Gibco, Gaithersburg, MD, United States) containing 10% fetal bovine serum (FBS) (BI, Israel) and 1% penicillin-streptomycin (Beyotime, Nantong, China) at 37°C and 5% CO_2_. Insulin-transferrin-sodium selenite media supplement (ITS) (Sigma, St. Louis, MO, United States) was added to the culture medium for 14 days after cell attachment to induce differentiation into chondrogenic ATDC5 cells according to a previous method (Mengqi Zhang et al., 2020).

### 2.2 Cell Counting Kit-8 Assay

Bilobalide (purity ≥ 98%) was purchased from Chengdu Must Bio-technology Co., Ltd. (Chengdu, China). Requirements considered to be relevant in guidelines for best practice in natural products pharmacological research have been taken into account (Heinrich et al., 2020). Viable cell proliferation was determined using CCK-8 (APExBIO, Houston, TX, United States). Cells were seeded in 96-well plates and treated with 10 ng/ml IL-1β (Novoprotein, China) and bilobalide (0, 7.5, 15, 30, 60, and 120 μM) for 12 or 24 h. Different concentrations of bilobalide dissolved in dimethylsulfoxide (DMSO) were added to the medium, with the final concentration of DMSO not exceeding 0.05%. Following that, 10 μl of CCK-8 solution was added. After 30 min, a multi-function microplate reader (BioTEK, Winooski, VT, United States) was used to measure the absorbance at 450 nm.

### 2.3 Dansylcadaverine Staining

Dansylcadaverine (MDC) staining (Solarbio, China) was used to observe the formation of autophagosomes as a marker of autophagy. The MDC staining procedure was based on a previous study (Ma et al., 2021). Briefly, cells were cultured in a 6-well plate with 10 ng/ml IL-1β and 60 μM bilobalide for 24 h before being fixed with 4% paraformaldehyde for 15 min. After removing the fixative, the cells were washed with PBS and incubated with MDC dye for 60 min. Autophagosomes were observed using a fluorescence microscope (Leica, Germany).

### 2.4 Transmission Electron Microscopy

Autophagic vesicles were observed using transmission electron microscopy. Chondrocytes treated with 10 ng/ml IL-1β and 60 μM bilobalide were collected. The chondrocytes were fixed with 2.5% glutaraldehyde at 4°C for 24 h, then dehydrated step by step using ethanol. Following that, the samples were washed thoroughly with PBS and embedded in resin. The samples were then sectioned and stained with 2% uranyl acetate aqueous solution for 1 h in the dark. Images were captured using an H-7650 electron microscope (Hitachi, Japan).

### 2.5 Immunofluorescence Staining

Cells were seeded in a confocal Petri dish (35 mm), then chondrocytes were cultured with or without 10 ng/ml IL-1β and 60 μM bilobalide for 24 h. The cells were then fixed with 4% paraformaldehyde for 15 min, washed with PBS, and permeabilized with .5% Triton X-100 for 15 min. Following that, the chondrocytes were blocked with 3% BSA for 30 min before being incubated with the SIRT1 antibody (ABclonal, Wuhan, China) (1:200) at 4°C overnight. Next, the cells were incubated with the Alexa 488 Goat Anti-Rabbit IgG (H+L) antibody (APExBIO) at room temperature for 1 h. The cell nuclei were counter-stained with DAPI (Beyotime) for 10 min. Images were captured using a fluorescence microscope (Leica).

### 2.6 Western Blotting

Total protein was extracted from rat cartilage and ATDC5 chondrocytes using Radio Immunoprecipitation Assay (RIPA) (Beyotime) containing protease and phosphatase inhibitors (MedChemExpress, New Jersey, United States). Protein concentrations were determined using a Bicinchoninic acid (BCA) protein assay kit (Beyotime). Equal quantities of total proteins were separated by Sodium Dodecyl Sulfate Polyacrylamide Gel Electrophoresis (SDS-PAGE) and transferred to a polyvinylidene difluoride membrane. After blocking with 5% bovine serum albumin (BSA) for 1 h, the membrane was incubated with the following primary antibodies overnight at 4°C: iNOS (1:1,500, ABclonal), COX-2 (1:2,000, ABclonal), MMP13 (1:2,000, ABclonal), GAPDH (1:2,000, ABclonal), p-mTOR (1:500, Wanleibio, China), mTOR (1:2,000, ABclonal), LC3 (1:2,000, Proteintech, China), p62 (1:2,000, ABclonal), p-AMPK (1:2,000, ABclonal), AMPK (1:2,000, ABclonal), and SIRT1 (1:1,000, ABclonal). Following that, the membrane was incubated with secondary antibodies (1:3,000, ZSGB-BIO, China) for 1 h. The ECL buffer (Vazyme, China) was added onto the membrane, and protein bands were visualized using the Tannon automatic gel image analysis system (China). The relative expression level of the protein was analyzed using the ImageJ software. The inhibitors, autophagy inhibitor chloroquine (CQ), AMPK inhibitor compound C, and SIRT inhibitor Selisistat (EX-527), were all purchased from MedChemExpress (United States).

### 2.7 Quantitative Real-Time Polymerase Chain Reaction Analysis

Total RNA was extracted using the RNA simple total RNA kit (Tiangen, China) according to the manufacturer’s instructions. Reverse transcription was performed using the ReverTra Ace qPCR RT Master Mix with gDNA Remover (TOYOBO, Japan). Complementary DNA (cDNA) was amplified using the ChamQ Universal SYBR qPCR Master Mix (Vazyme). The expression levels of the target genes were evaluated using a relative quantification approach (2^−ΔΔCT^ method) against β-actin levels. The forward and reverse primer sequences of all genes are listed in Table 1.

**TABLE 1:** The primer sequences used in qPCR assay.

Gene	Forward primer (5′-3′)	Reverse primer (5′-3′)
β-actin	5′-GTG​ACG​TTG​ACA​TCC​GTA​AAG​A-3′	5′-GCC​GGA​CTC​ATC​GTA​CTC​C-3′
Atg3	5′-ACA​CGG​TGA​AGG​GAA​AGG​C-3′	5′-TGG​TGG​ACT​AAG​TGA​TCT​CCA​G-3′
Atg4	5′-GCT​GGT​ATG​GAT​TCT​GGG​GAA-3′	5′-TGG​GTT​GTT​CTT​TTT​GTC​TCT​CC-3′
Atg5	5′-TGT​GCT​TCG​AGA​TGT​GTG​GTT-3′	5′-ACC​AAC​GTC​AAA​TAG​CTG​ACT​C-3′
Atg6	5′-ATG​GAG​GGG​TCT​AAG​GCG​TC-3′	5′-TGG​GCT​GTG​GTA​AGT​AAT​GGA-3′
Atg7	5′-TCT​GGG​AAG​CCA​TAA​AGT​CAG​G-3′	5′-GCG​AAG​GTC​AGG​AGC​AGA​A-3′
Atg9	5′-CCG​AGG​GGA​GCA​AAT​CAC​C-3′	5′-TAG​TCC​ACA​CAG​CTA​ACC​AGG-3′
Atg12	5′-TGA​ATC​AGT​CCT​TTG​CCC​CT-3′	5′-CAT​GCC​TGG​GAT​TTG​CAG​T-3′
Atg13	5′-CCA​GGC​TCG​ACT​TGG​AGA​AAA-3′	5′-AGA​TTT​CCA​CAC​ACA​TAG​ATC​GC-3′
Atg16	5′-GCC​CAG​TTG​AGG​ATC​AAA​CAC-3′	5′-CTG​CTG​CAT​TTG​GTT​GTT​CAG-3′

### 2.8 ACLT-Induced Rat Model of Post-Traumatic OA

Forty male Sprague-Dawley rats (12-weeks-old, 300–350 g) were purchased from Changchun Yisi Experimental Animal Technology Co., Ltd. (License number: SCXK-2018-007) (Changchun, China) and kept in a controlled environment (light/dark, 12/12 h; temperature 23 ± 1°C). The rats were acclimated for 1 week before experiments were carried out. The rats were randomly divided into five groups: control (sham operation and treated with a salt solution), OA (ACLT surgery to induce OA), celecoxib (2.86 mg/kg/day celecoxib [Pfizer Pharmaceutical, New York, United States] (Panahifar et al., 2014) was administered as a positive control), 5 mg/kg + Bilobalide (the OA rats treated with 5 mg/kg/day of bilobalide), and 10 mg/kg + Bilobalide (the OA rats treated with 10 mg/kg/day of bilobalide), with eight rats in each group. The ACLT-induced PTOA models for rats and animal postoperative care were established according to previous studies (Ma et al., 2020). Rats in the 5 mg/kg + Bilobalide and 10 mg/kg + Bilobalide groups received oral bilobalide daily for 6 weeks. The bilobalide dosage was based on previous studies (Heng Zhang et al., 2020; Zhao et al., 2021). After 6 weeks of treatment, the rats in each group were euthanized by intraperitoneal injection of a lethal dose of ether, and their knee joints and blood samples were taken. The blood was centrifuged at 1,000 g for 20 min at room temperature. The supernatant was collected and stored at −80°C for further analysis. The animal surgery procedure and treatments used in this study were approved by The Laboratory Animal Welfare and Ethics Committee of Northeast Agricultural University (#NEAU-2021-03-1157-6). Every effort was made to minimize animal suffering and reduce the number of animals used.

### 2.9 Animal Behavior Tests

#### 2.9.1 Mechanical Sensitivity

After ACLT surgery, rats were subjected to weekly behavioral tests. Mechanical sensitivity was assessed by measuring the withdrawal thresholds of both hind paws in response to the application of von Frey filaments using the up-down method, as previously described (Katri et al., 2019).

#### 2.9.2 Vocalizations Evoked by Extension of the Knee

The knee extension test was performed for the right leg by starting with the knee in the resting position (slightly flexed). The knee was extended while the thigh was held. The number of vocalizations that occurred during the five extensions was recorded (Im et al., 2010; Piel et al., 2014). After the habituation procedure, mechanical sensitivity and vocalizations evoked by extension of the knee were assessed before surgery and up to 6 weeks post-surgery. The behavioral tests were evaluated by two independent researchers (LL and YY). Any disagreements between the researchers were resolved by a third researcher (LG.).

### 2.10 Histological Assessments

All rats were killed 6 weeks after ACLT. Knee joint samples were collected, fixed in 4% paraformaldehyde for 24 h, decalcified in 10% EDTA at 4°C for several days, embedded in paraffin then sectioned into 4-lm-thick sections. For further histological analyses, hematoxylin-eosin (HE) and toluidine blue (TB) staining were performed on the sections. Histopathological features were semi-quantitatively scored according to the Osteoarthritis Research Society International (OARSI) grading system (Pritzker et al., 2006).

### 2.11 ELISA Detection of Rat Osteoarthritis Biomarkers

The levels of C-telopeptides of type II collagen (CTX-II), Cartilage oligomeric matrix protein (COMP), and Collagenase cleavage neopeptide (C2C) in rat serum were determined with ELISA. The ELISA kit was purchased from Shanghai Enzyme-linked Biotechnology Co., Ltd. (Mlbio, China). ELISA was performed according to the manufacturer’s instructions. Three replicate wells were used for each set of samples and the average OD value was obtained. The OD values at 450 nm were measured using a multi-function microplate reader (BioTEK). All experiments were conducted in the Heilongjiang Key Laboratory of Animals Disease Pathogenesis and Comparative Medicine.

### 2.12 Statistical Analysis

All statistical analyses were performed using the SPSS software (version 19.0 for Windows, Chicago, IL, United States). Results were presented as mean ± standard (SD) deviation. The data were evaluated for Gaussian distribution using the Kolmogorov–Smirnov test. All data had normal distribution and were analyzed with parametric statistics (one-way ANOVA, two-way ANOVA, paired two-tailed Student’s t-test, and unpaired two-tailed Student’s t-test). *p* < .05 was considered statistically significant.

## 3 Results

### 3.1 Effect of Bilobalide on ATDC5 Chondrocyte Viability

**FIGURE 1:**
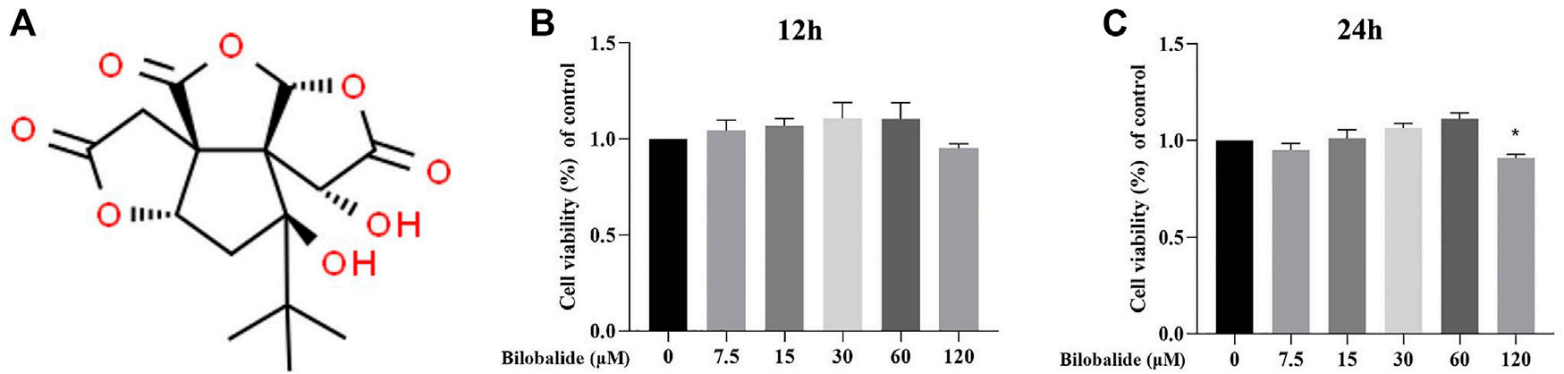
The effect of bilobalide on the viability of ATDC5 chondrocytes. **(A)** Chemical structure of bilobalide. **(B,C)** Bilobalide cytotoxicity on chondrocytes at 12 and 24 h. All data were presented as mean ± SD (*n* = 5). *p < .05 indicates significant differences between different groups compared to the control group (Statistical analysis using one-way ANOVA and paired two-tailed Student’s t-test).

The molecular structure of bilobalide is shown in Figure 1A. Bilobalide intervention for 12 h had limited cytotoxicity on ATDC5 chondrocytes (Figure 1B). However, after 24 h of intervention, bilobalide at a concentration of 120 mΜ significantly reduced ATDC5 chondrocytes viability (Figure 1C). When cells were stimulated with bilobalide for 24 h, the activity of the cells increased at a concentration of 7.5–60 μM. Therefore, bilobalide was used at 15, 30, and 60 μM in subsequent experiments, with a treatment time of 24 h.

### 3.2 Bilobalide Inhibited IL-1β-Induced Pro-inflammatory Cytokines and MMP13 Production in ATDC5 Chondrocytes

**FIGURE 2:**
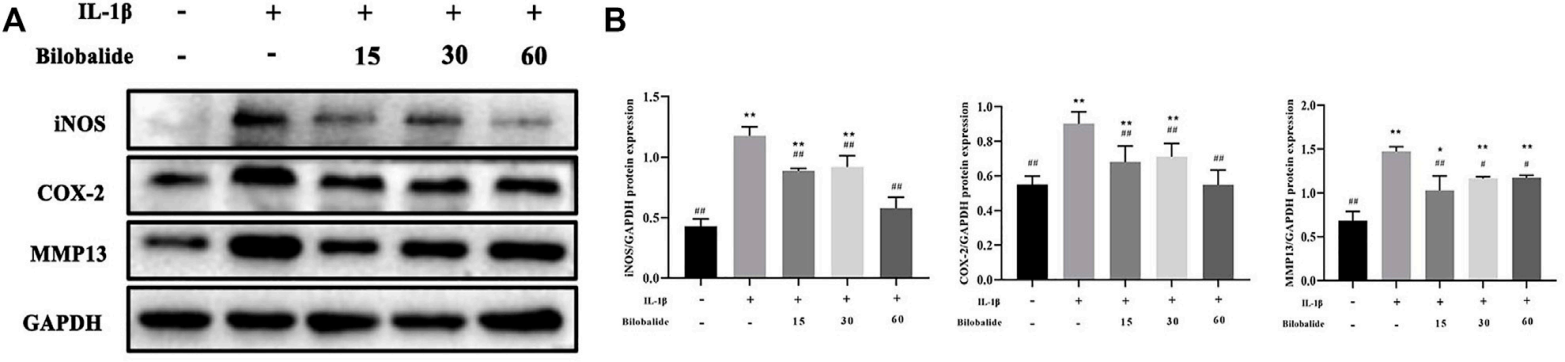
Influence of bilobalide on the IL-1β-induced inflammatory reaction in ATDC5 chondrocytes. **(A,B)** Western blot analysis of iNOS, COX-2, and MMP13 protein levels in chondrocytes. All data were presented as mean ± SD (*n* = 3). *p < .05 and **p < .01 compared to the control group; ^#^ *p* < .05 and ^##^ *p* < .01 compared to the IL-1β group (Statistical analysis using one-way ANOVA and paired two-tailed Student’s t-test).

Chondrocytes were treated with 10 ng/ml IL-1β in the presence or absence of bilobalide for 24 h. In chondrocytes, 10 ng/ml IL-1β stimulation caused an abnormal increase in inducible nitric oxide synthase (iNOS) and cyclooxygenase-2 (COX-2) inflammatory factors (Figures 2A,B). However, bilobalide could antagonize those effects. In addition, bilobalide significantly inhibited the expression of the MMP13 protein (*p* < .05). In ATDC5 chondrocytes induced by IL-1β, 60 μM bilobalide intervention had the greatest inhibitory effect on iNOS and COX-2. The above results indicated that bilobalide could protect ATDC5 chondrocytes from IL-1β-induced inflammation by down-regulating the expression of iNOS, COX-2, and MMP13.

### 3.3 Bilobalide Initiates Autophagy in IL-1β-Induced ATDC5 Chondrocytes

**FIGURE 3:**
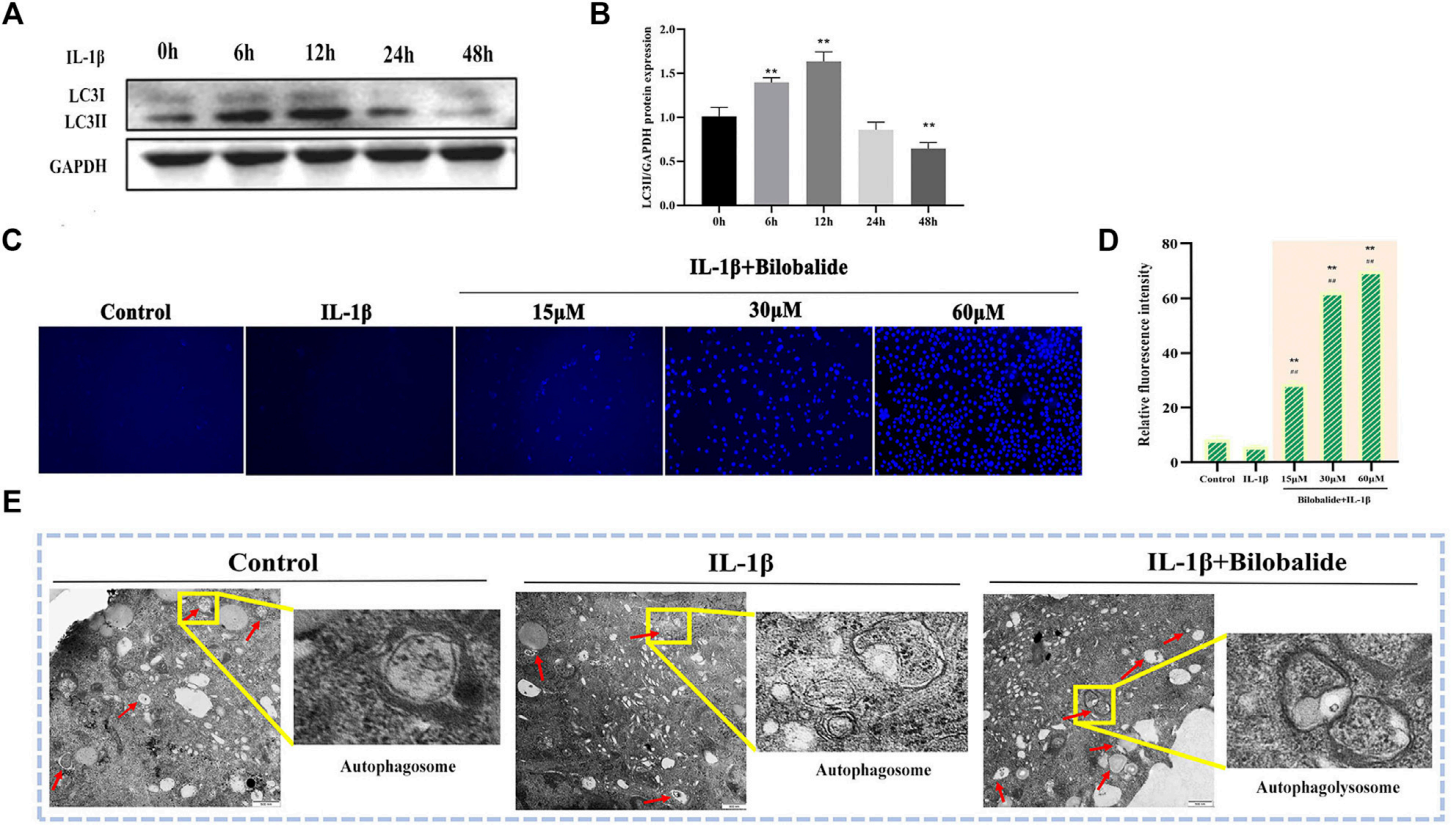
Bilobalide induces autophagy in IL-1β-induced ATDC5 chondrocytes. **(A,B)** Western blot analysis of LC3II protein expression after IL-1β induced cartilage for different periods. **(C,D)** The number of autophagosomes was observed using MDC dye under a fluorescence microscope. Magnification ×10. **(E)** Autophagic vesicles were observed using transmission electron microscopy. Magnification ×3000. All data were presented as mean ± SD (*n* = 3). ** *p* < 0.01 compared to the control group; #*p* < 0.05 and ##*p* < 0.01 compared to the IL-1β group (Statistical analysis using one-way ANOVA and paired two-tailed Student’s t-test).

In order to exclude autophagy induced by serum starvation (Rong et al., 2021), the DMEM/F12 medium containing 10% fetal bovine serum was consistently used in this study. Because IL-1β induction may also affect autophagy (Ge et al., 2018), the expression of the autophagy marker protein LC3II was examined at different 10 ng/ml IL-1β induction times. The results showed that after chondrocytes were exposed to 10 ng/ml IL-1β for 24 h, the expression of LC3Ⅱ protein was significantly reduced (*p* < .05) and there was a decrease in chondrocyte autophagy (Figures 3A,B). Therefore, based on the above experimental results and combined with the previous CCK-8 results, 10 ng/ml IL-1β was selected to treat cells for 24 h in subsequent experiments.

The results of MDC staining showed that the number of autophagosomes in the IL-1β + bilobalide group increased in a dose-dependent manner compared to the control group (*p* < .05) (Figures 3C,D). In order to verify the initiation of autophagy, transmission electron microscopy was used to observe autophagic vesicles (Figure 3E). When IL-1β and bilobalide were both added, autophagic vesicles were abundant, including autophagosome with double-layer membranes and autophagolysosome with single-layer membranes, compared to treatment with 10 ng/ml IL-1β alone.

### 3.4 Bilobalide Inhibited IL-1β-Induced ATDC5 Chondrocyte Inflammation by Restoring Autophagy

**FIGURE 4:**
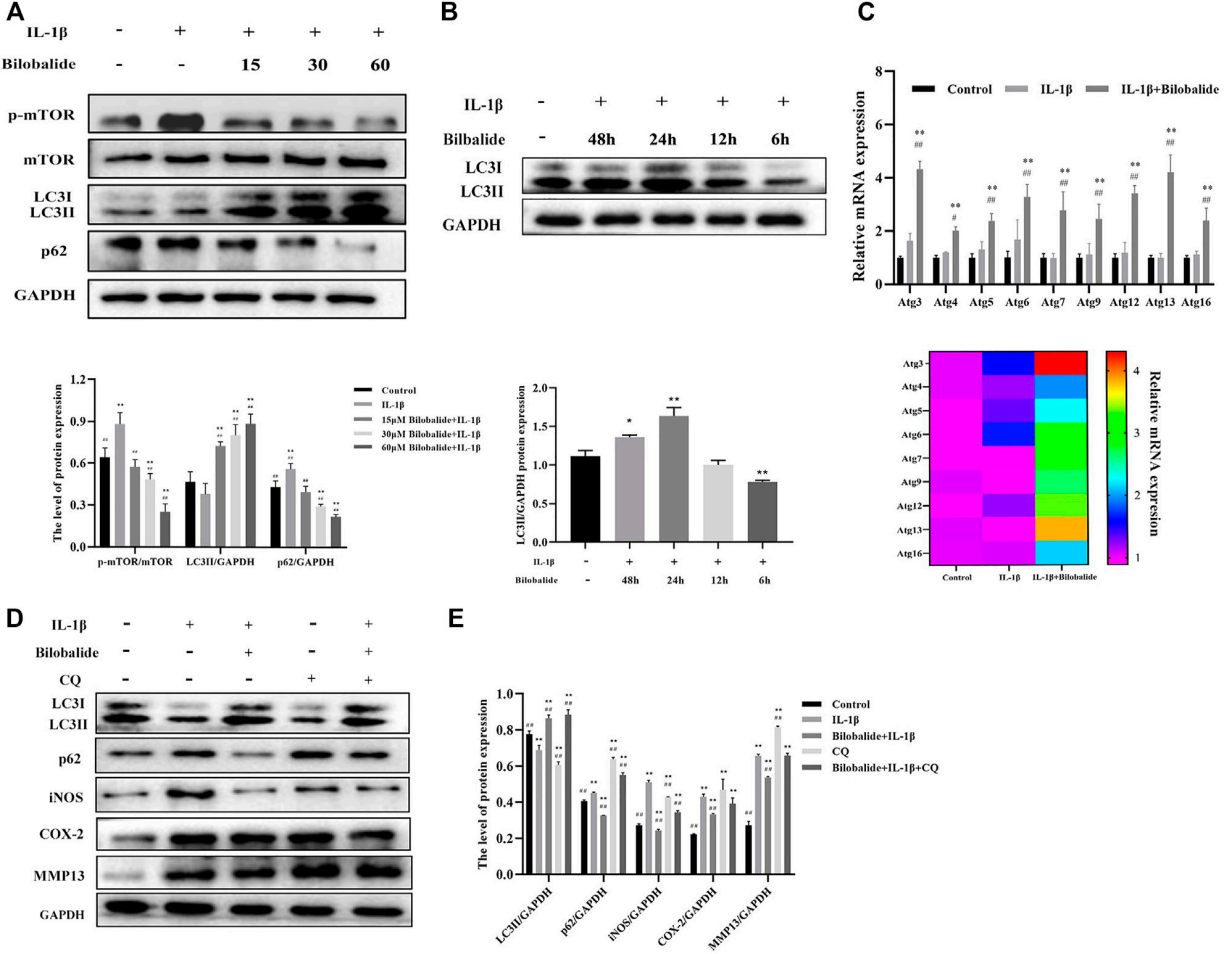
The effect of bilobalide on autophagy in ATDC5 chondrocytes induced by IL-1β. **(A)** Western blot analysis of mTOR, p-mTOR, LC3II, and p62 protein expression after treatment with 10 ng/ml IL-1β and different concentrations bilobalide for 24 h. **(B)** The effect of 60 μM bilobalide with different incubation periods in the presence of 10 ng/ml IL-1β on autophagy was determined through the LC3II protein using western blot. **(C)** The expression levels of certain autophagy-related genes (Atg genes) were evaluated using qPCR. **(D,E)** Chondrocytes were pretreated with 30 μM CQ. The protein levels of LC3II, p62, COX-2, and MMP13 were examined by Western blot. All data were presented as mean ± SD (*n* = 3). *p < .05 and **p < .01 compared to the control group; ^#^ *p* < .05 and ^##^ *p* < .01 compared to the IL-1β group (Statistical analysis using one-way ANOVA, two-way ANOVA, paired two-tailed Student’s t-test, and unpaired two-tailed Student’s t-test).

Next, the expression of p-mTOR, mTOR, LC3II, and p62 in ATDC5 chondrocytes was evaluated (Figure 4A). Compared to the control group, the expression of p-mTOR and p62 increased after IL-1β stimulation (*p* < .05), while the expression of LC3II significantly decreased (*p* < .05). However, after the addition of bilobalide, the expression of p-mTOR and p62 decreased in a dose-dependent manner, while LC3II expression displayed a dose-dependent increase. These findings demonstrated that bilobalide promoted autophagy in a dose-dependent manner.

Following that, a kinetic experiment was performed (Figure 4B). Chondrocytes were treated with 10 ng/ml IL-1β and 60 μM bilobalide at the same time for 6–48 h to determine the expression of LC3II. The expression of LC3II significantly increased after 24 h of treatment (*p* < .05). Autophagy induced by IL-1β in the presence of bilobalide was most prominent within 24 h. In addition, qPCR results showed that the relative level of the Atg genes significantly increased compared to the IL-1β group after treatment with 10 ng/ml IL-1β and 60 μM bilobalide for 24 h (*p* < .05) (Figure 4C).

### 3.5 Autophagy is Required for Bilobalide’s Anti-Inflammation Activity

In order to better understand the mechanism of autophagy and inflammation induced by bilobalide in ATDC5 chondrocytes, we analyzed the expression of LC3II, p62, iNOS, COX-2, and MMP13 of the 60 μM bilobalide treated group after intervention with 30 μM CQ (Figures 4D,E). The chondrocytes were divided into five groups: (I) control, (II) IL-1β, (III) Bilobalide + IL-1β, (IV) CQ, and (V) Bilobalide + IL-1β + CQ. Groups IV and V were pretreated with CQ for 1 h, then incubated with bilobalide for 24 h. The combined action of IL-1β and bilobalide resulted in significantly increased expression of the LC3II protein (*p* < .05), while the expression of the p62 protein was significantly inhibited (*p* < .05). After adding the CQ inhibitor alone, the expression of the LC3II protein was significantly reduced (*p* < .05), while the expression of the p62 protein was significantly increased (*p* < 0.05). Interestingly, the expression of LC3II and p62 in the bilobalide + IL-1β + CQ group increased compared to the bilobalide + IL-1β group. We hypothesized that CQ blocked the fusion of autophagic vesicles and lysosomes, thereby inhibiting the degradation of the LC3II and the p62 protein. In addition, bilobalide significantly inhibited the up-regulation of iNOS, COX-2, and MMP3 expression in chondrocytes induced by IL-1β, while the addition of CQ reversed the decrease in iNOS, COX-2, and MMP3 proteins caused by bilobalide.

### 3.6 Bilobalide Promotes Chondrocyte Autophagy by Regulating AMPK/SIRT1/mTOR Pathway

**FIGURE 5:**
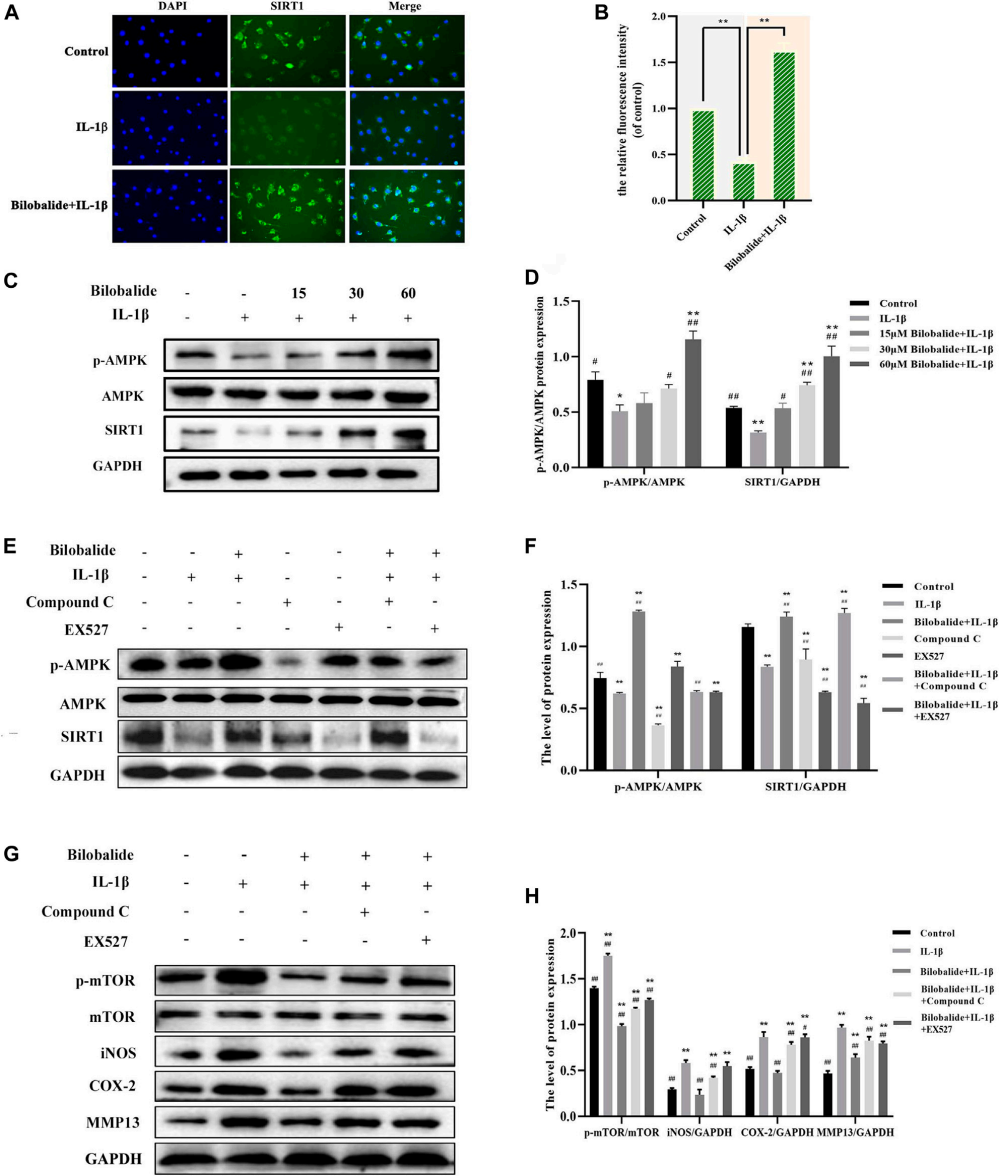
The effect of bilobalide on the AMPK/SIRT1/mTOR signaling pathway in ATDC5 chondrocytes induced by IL-1β. **(A,B)** The fluorescence intensity of the Sitr1 protein was detected by immunofluorescence. **(C,D)** Western blot analysis of AMPK, p-AMPK, and SIRT1 protein expression after treating the cells with IL-1β and different concentrations bilobalide for 24 h **(E,F)** Western blot analysis of AMPK, p-AMPK, and SIRT1 protein expression after chondrocytes were pretreated with the AMPK inhibitor compound C and the SIRT1 inhibitor EX-527. **(G,H)** Western blot analysis of mTOR, p-mTOR,iNOS, COX-2, and MMP13 protein expression after the addition of compound C or EX-527. All data were presented as mean ± SD (*n* = 3). *p < .05 and **p < .01 compared to the control group; ^#^ *p* < .05 and ^##^ *p* < .01 compared to the IL-1β group (Statistical analysis using one-way ANOVA, two-way ANOVA, paired two-tailed Student’s t-test, and unpaired two-tailed Student’s t-test).

The expression of the SIRT1 protein was detected by immunofluorescence. Compared to the control group, SIRT1 expression was significantly increased after 10 ng/ml IL-1β and 60 μM bilobalide treatment (*p* < .05) (Figures 5A,B). Western blot analysis revealed that the expression of p-AMPK and SIRT1 increased in a dose-dependent manner compared to the control group (*p* < .05) (Figures 5C,D). Chondrocytes were pretreated with compound C and EX-527 to verify the activation of AMPK and SIRT1 (Figures 5E,F). In the presence of 60 μM bilobalide and 10 ng/ml IL-1β, pretreatment with compound C significantly reduced phosphorylation of AMPK (*p* < .05), while EX-527 significantly inhibited the expression of SIRT1 (*p* < .05). After 60 µM bilobalide was added, the expression of p-AMPK and SIRT1 in chondrocytes significantly increased (*p* < .05), but the degree of increase was decreased by the addition of compound C or EX-527, respectively. This suggested that bilobalide activated the AMPK/SIRT1 signaling pathway. In order to further explore the role of AMPK/SIRT1 in autophagy signaling, compound C or EX-527 were used to determine the protein levels of mTOR, p-mTOR, iNOS, COX-2, and MMP13 (Figures 5G,H). The results revealed that in the presence of bilobalide and IL-1β, the expression of p-mTOR, iNOS, COX-2, and MMP13 were significantly reduced (*p* < .05). However, the addition of compound C or EX-527 improved the reduction of p-mTOR, iNOS, COX-2, and MMP13 (*p* < .05). These results suggested that the AMPK/SIRT1 pathway regulates the level of phosphorylation of mTOR, thereby modulating downstream cascades related to autophagy.

### 3.7 Bilobalide Attenuates ACLT-Induced Post-Traumatic OA in Rats *via* AMPK/SIRT1/mTOR Pathway

**FIGURE 6:**
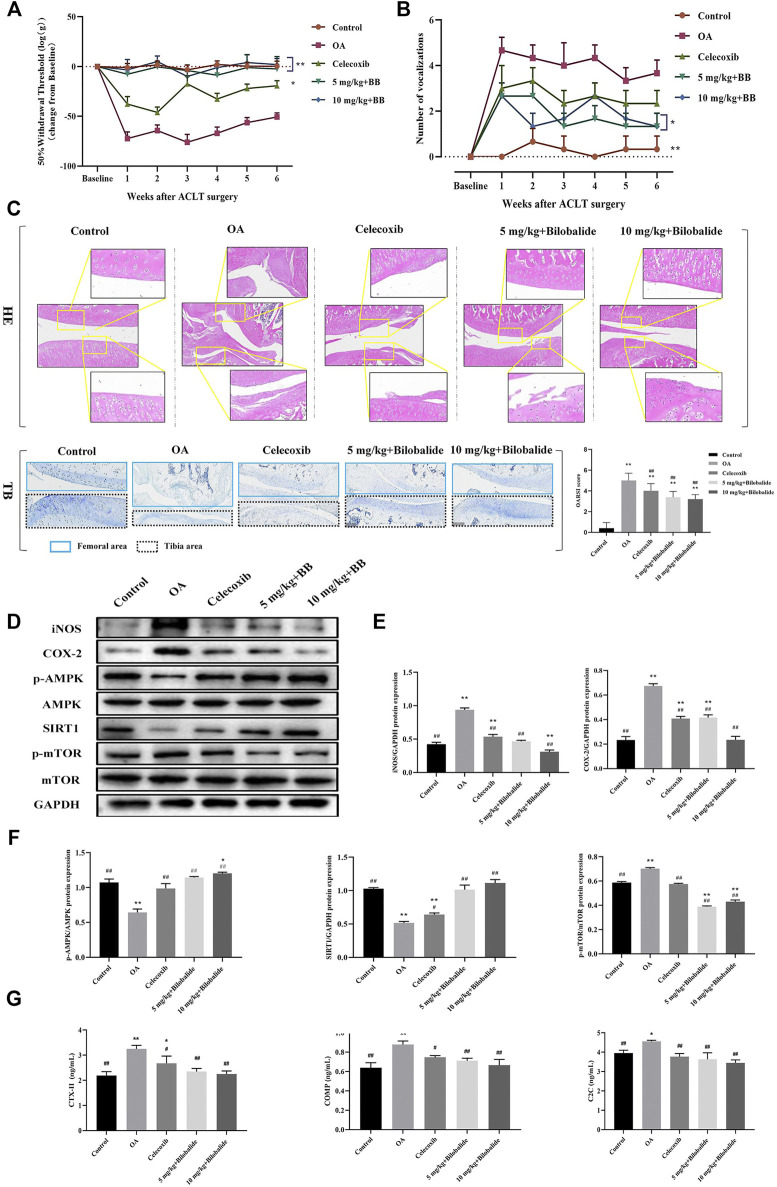
Bilobalide attenuates ACLT-induced PTOA in rats via AMPK/SIRT1/mTOR pathway. **(A)** Mechanical sensitivity. **(B)** Vocalizations evoked by extension of the knee. **(C)** Representative images of the knee joint stained with hematoxylin-eosin (HE) and toluidine blue (TB) and OARSI scores after 6 weeks of treatment. Magnification ×10. **(D–F)** Western blot analysis of AMPK/SIRT1/mTOR pathway related proteins expression in rat cartilage. **(G)** The levels of ECM degradation biomarkers CTX-II, COMP, and C2C in the serum of rats after bilobalide intervention were determined using ELISA. All data were presented as mean ± SD (*n* = 3). *p < .05 and **p < .01 compared to the control group; ^#^ *p* < .05 and ^##^ *p* < .01 compared to the IL-1β group (Statistical analysis using one-way ANOVA and paired two-tailed Student’s t-test).

Compared to the control group, the mechanical sensitivity of the rats was significantly reduced within 6 weeks of establishing the model, as seen in Figure 6A. In comparison to the OA group, the mechanical sensitivity of rats was significantly increased after the administration of 5 mg/kg and 10 mg/kg bilobalide. In the knee extension test, the number of vocalizations was also significantly reduced in rats after 6 weeks of bilobalide administration (Figure 6B).

The representative HE and TB images and OARSI scores of the knee joints of each group are shown in Figure 6C. The cartilage structure and layers of the control group were clear and complete, the chondrocytes were arranged neatly, and TB was evenly stained. The articular cartilage in the OA group was severely damaged, with obvious cracks appearing in the tibia and femur. Furthermore, the chondrocytes were vacuolated and arranged in clusters, and the intensity of TB staining was reduced. In the celecoxib group, the cartilage surface was rough, the surface of the femur was slightly rough, the surface chondrocytes were destroyed, the surface of the tibia was severely damaged, the inflammatory cells infiltrated to the middle layer, and the cell arrangement was disordered. Compared to the OA group, the OARSI score of the celecoxib group was significantly lower (*p* < .05). After treatment with bilobalide, the cartilage of the knee joint of rats was protected; the surface roughness of the femur was significantly reduced and the cell arrangement was relatively in order. In the 5 mg/kg + Bilobalide group a part of the tibia was still infiltrated by inflammation; the chondrocytes were disordered; however, the intensity of TB staining was stronger than the OA group. In the 10 mg/kg + Bilobalide group, chondrocyte hypertrophy and vacuolization were observed in the femoral area, and there was no obvious loss of chondrocytes; compared to the OA group, the OARSI score of rats after bilobalide administration was significantly reduced (*p* < .05).

As shown in Figures 6D,E, 6 weeks after ACLT surgery, the expression of inflammatory factors iNOS and COX-2 in the OA group was significantly increased (*p* < .05), while bilobalide inhibited the expression of iNOS and COX-2. Notably, 10 mg/kg bilobalide had the most significant therapeutic effect. We observed that compared with the OA group, the expression of p-AMPK and SIRT1 was significantly increased (*p* < .05) after bilobalide administration, while the expression of p-mTOR was inhibited by bilobalide (Figures 6D,F). This indicates that Bilobalide attenuates cartilage iNOS and COX-2 levels in PTOA rats through the AMPK/SIRT1/mTOR pathway.

The type II collagen degradation products CTX-II and C2C, and non-collagen COMP in ECM are OA biomarkers for evaluating cartilage damage. As shown in Figure 6G, the levels of CTX-II, COMP, and C2C in the serum of rats in the OA group were significantly increased compared to the control group (*p* < .05). The celecoxib group, as a positive control, effectively reduced the expression of three biomarkers in the serum compared to the OA group (*p* < .05). After 6 weeks of bilobalide intervention, the levels of CTX-II, C2C, and COMP in the serum of rats were significantly reduced (*p* < .05). Furthermore, 10 mg/kg bilobalide had a better effect than other groups. These findings demonstrated that bilobalide could effectively reduce the degradation of ECM in rats, thereby protecting the joints.

## 4 Discussion

*Ginkgo biloba* L. extract has anti-inflammatory properties (Ilieva et al., 2004; Ye et al., 2019) and its sesquiterpene lactone compound bilobalide may have significant pharmacological effects (Goldie and Dolan, 2013). A large number of studies have confirmed that IL-1β is a pro-inflammatory factor that plays a key role in the pathological process of OA (Wang and He, 2018; Jenei-Lanzl et al., 2019). IL-1β induced increased OA-related protein expressions in a time and concentration dependent manner (Tu et al., 2019). The stimulation of IL-1β (10 ng/ml) for 24 h could induce inflammation in chondrocytes (Wang et al., 2019; Pei et al., 2021). IL-1β has been shown to induce the overproduction of iNOS and COX-2, which is directly relevant to the secretion of nitric oxide (NO) and prostaglandin E2 (PGE2) (Sheu et al., 2015). In our study, the expression of iNOS, COX-2 and MMP13 increased after 10 ng/ml IL-1β induced chondrocytes, which proved that 10 ng/ml IL-1β successfully induced chondrocyte inflammation model *in vitro*. In addition, bilobalide inhibited the expression of the iNOS, COX-2, and MMP13 proteins. In the rat PTOA model established by ACLT, bilobalide induced and inhibited the expression of iNOS and COX-2 in rat cartilage tissue. These results indicated that bilobalide inhibited inflammation and slowed down the degradation of chondrocyte ECM.

Pro-inflammatory cytokines and autophagy play a key role in the pathophysiology of diseases, and there is evidence to prove the cross-talk effect between autophagy and pro-inflammatory cytokines (Ge et al., 2018). IL-1β was demonstrated to trigger autophagosome formation, and they may induce autophagy as part of a negative feedback loop to limit the inflammatory response, and promote cytokine-mediated anti-microbial defense (Zhang et al., 2015). Autophagic processes can orchestrate the transcription, processing, and production of proinflammatory cytokines, serving as a negative feedback loop for the modulation of inflammatory as well as immune responses. Therefore, in order to clarify the optimal conditions under which IL-1β-induced chondrocyte autophagy cannot be detected, we set different IL-1β induction time under the premise of determining the concentration of 10 ng/ml IL-1β, and observe the influence of IL-1β time gradient on autophagy. Similarly, Rong et al. used LPS (0.5 μg/ml) to induce the RAW264.7 cell inflammation model, and detected the autophagy induced by LPS at different times (0–48 h) by Western blot, and found that the expression of LC3/p62 protein was reduced after 24 h of induction (Rong et al., 2021). It has been reported that compared with normal chondrocytes, rat chondrocytes treated with 10 ng/ml IL-1β for 24 h have observed a reduction in autophagy levels (Yan et al., 2021). Interestingly, we found that IL-1β (10 ng/ml) induced ATDC5 chondrocytes for 24 h, and the autophagy level was significantly reduced, which is consistent with previous studies (Zhou et al., 2021). We determined that 10 ng/ml IL-1β induced a significant decrease in the autophagy intensity of chondrocytes for 24 h.

Recent studies believe that autophagy is a key regulator of OA and a potential therapeutic target (Valenti et al., 2021; Tian et al., 2021). The production of LC3II is a common sign of autophagy maturation, and the scaffold protein p62 is the substrate protein of autophagy, which mainly accumulates substances that need to be degraded in the cell, reflecting the degree of autophagy degradation (Tanida, 2011). We found that the autophagosomes after the co-treatment of IL-1β and bilobalide increased significantly, indicating that chondrocyte autophagy was initiated. LC3II protein expression was significantly increased after bilobalide treatment, and phosphorylated mTOR and p62 proteins were significantly reduced, indicating that bilobalide induced chondrocyte autophagy by inhibiting the mTOR pathway, and the substances in autophagy were successfully degraded. Our results showed that the co-treatment of bilobalide and IL-1β significantly up-regulated the expression of autophagy-related genes (Atg genes). These results indicate that after mTOR phosphorylation is inhibited by upstream signals, the activated ULK1-Atg13-FIP200 complex participates in the formation of autophagic vesicles by activating Atg6, class III phosphatidylinositol 3-kinase and Atg14 complex (Yang and Klionsky, 2010). After autophagy is induced, LC3-I is bound to the highly lipophilic phosphatidylethanolamine (PE) by Atg7, Atg3, and Atg12-Atg5-Atg16L complex to produce LC3-II. Finally, PE promotes the integration of LC3-II into the lipid membrane to form autophagosomes. Among them, Atg9 played a key role in promoting the lipidation of Atg8 (Sawa-Makarska et al., 2020). Interestingly, We found that CQ effectively inhibited bilobalide-induced chondrocyte autophagy by inhibiting the degradation of LC3II and P62 protein. After CQ inhibited autophagy, the down-regulation of iNOS, COX-2 and MMP3 proteins induced by bilobalide was weakened. These findings indicated that autophagy is required for the protective effect of bilobalide in ATDC5 chondrocytes. This study discovered that the co-treatment with bilobalide and IL-1β increased the expression of p-AMPK and Srit1. However, the addition of compound C or EX-527 resulted in a lower increase in p-AMPK and Srit1. In the presence of bilobalide and IL-1β, the expression of p-mTOR, iNOS, COX-2, and MMP13 were significantly reduced, and the reduction of p-mTOR, iNOS, COX-2, and MMP13 were attenuated after the addition of compound C or EX-527. Previous studies have revealed that metformin has been shown to increase the phosphorylation level of AMPK and up-regulate SIRT1 protein expression, leading to increased chondrocyte autophagy and decreased catabolism (Wang et al., 2020), which is consistent with our findings. Furthermore, we validated that bilobalide regulates the AMPK/SIRT1 signaling pathway and reduces the expression of mTOR to activate autophagy and protect chondrocytes from damages.

The use of NSAIDs places a greater physical and financial burden on patients (Cooper et al., 2019). Therefore, it is particularly important to study natural compounds that have significant efficacy, few side effects, and can treat OA. Previous studies have shown that L-theanine can reduce the level of C2C in the serum of rats with OA induced by ACLT (Bai et al., 2020). Isorhamnetin inhibits the expression of COMP and CTX-II in MIA-induced OA rats and prevents cartilage damage (Tsai et al., 2019). In this study, we discovered that bilobalide had obvious health effects on OA. In the rat model of PTOA induced by ACLT, the pathological changes of cartilage were weakened after 6 weeks of oral administration of bilobalide, especially the protective effect on the femur. The serum levels of CTX-II, COMP, and C2C in the model group were significantly higher than the control group, indicating that degradation of type II collagen and proteoglycan in the cartilage ECM of PTOA rats led to the aggravation of OA. After bilobalide was administered, the levels of CTX-II, COMP, and C2C in the rat serum were significantly reduced, indicating that bilobalide inhibited ECM degradation and exerted chondroprotective effects. Furthermore, it was able to reduce joint pain in PTOA rats, reducing the symptoms of OA; this discovery is critical for future clinical studies.

**FIGURE 7:**
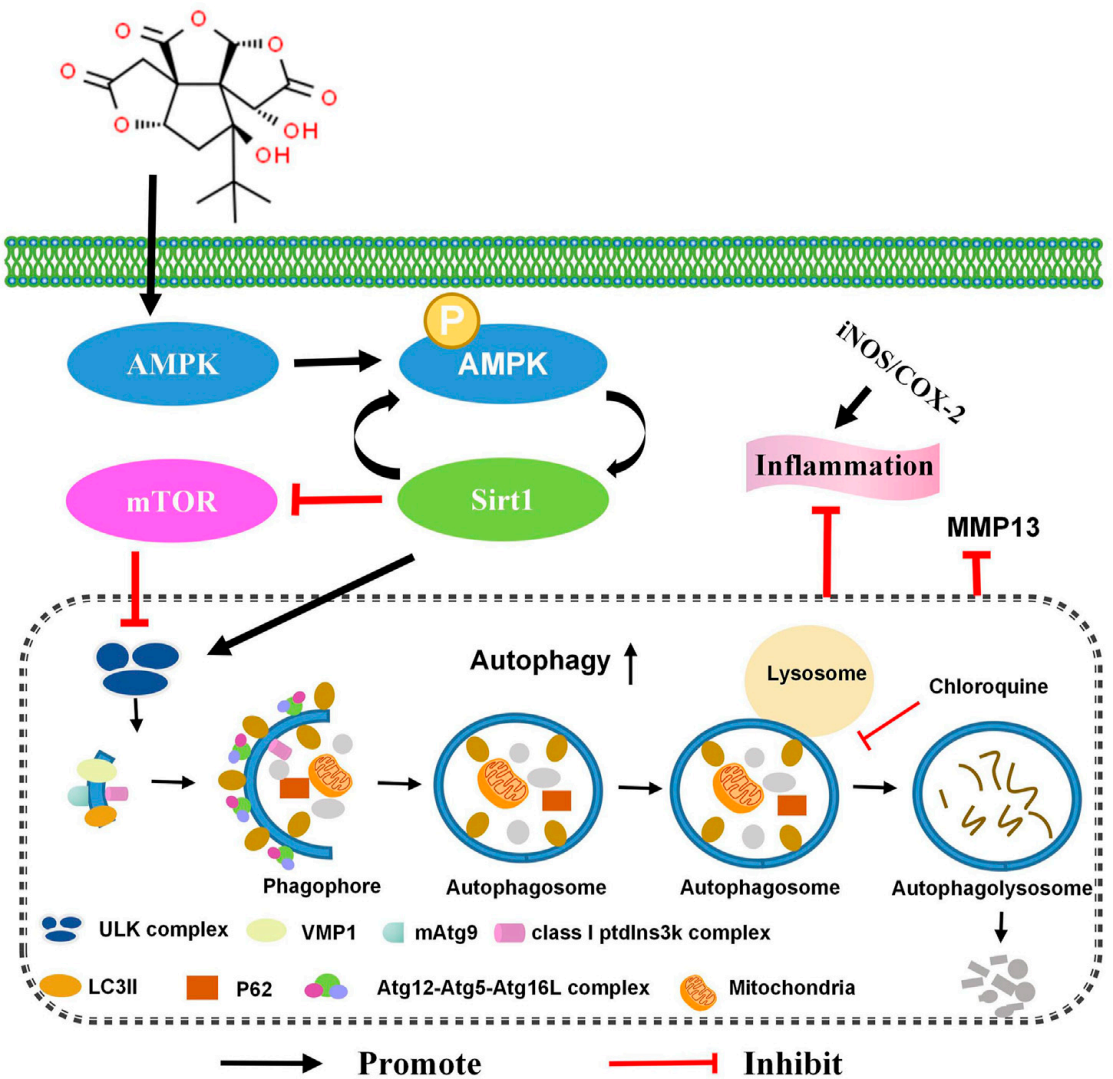
Bilobalide reduces IL-1β-induced ATDC5 chondrocyte inflammation by activating the AMPK/SIRT/mTOR pathway, leading to chondrocyte autophagy.

The long-term safety of *Ginkgo biloba* L. extract is unclear, which limits its clinical application (Mahadevan and Park, 2008). In addition, excessive consumption of Ginkgo leaf extracts has been reported to cause occasional adverse effects, including gastrointestinal disturbances, dizziness, allergic skin reactions, headaches, excessive bleeding, and anaphylaxis-like reactions (only with intravenous administration) (Vale, 1998; Benjamin et al., 2001; De Smet, 2002; Mahadevan and Park, 2008). We discovered that bilobalide, a unique constituent of *Ginkgo biloba* L., up-regulates chondrocyte autophagy flux and autophagy-related genes via the AMPK/SIRT1/mTOR signaling pathway. Furthermore, it reduces the levels of iNOS, COX-2, and MMP13, and has a protective effect on ATDC5 chondrocytes (Figure 7).

This study revealed that bilobalide has significant anti-inflammatory and anti-ECM degradation effects in ACLT-induced rat PTOA, suggesting that it could delay the progression of OA caused by cartilage pathology and damage. However, the study only focused on cartilage degeneration. More studies are therefore needed to clarify the effects of bilobalide on synovial inflammation and subchondral bone remodeling. Moreover, further *in vivo* research is required to establish the effectiveness bilobalide in the treatment of OA. Importantly, extensive pharmacological and toxicity studies are required for the treatment of OA with bilobalide before investigating the possibility of its clinical application. Nonetheless, Our results could provide new insights regarding the protective mechanism of bilobalide on articular cartilage.

## Data Availability

The original contributions presented in the study are included in the article/Supplementary Material, further inquiries can be directed to the corresponding author.

## References

[B1] Achete de Souza G. de Marqui S. V. Matias J. N. Guiguer E. L. Barbalho S. M. (2020). Effects of Ginkgo Biloba on Diseases Related to Oxidative Stress. Planta Med. 86 (6), 376–386. 10.1055/a-1109-3405 32097975

[B2] Asby D. J. Cuda F. Beyaert M. Houghton F. D. Cagampang F. R. Tavassoli A. (2015). AMPK Activation via Modulation of De Novo Purine Biosynthesis with an Inhibitor of ATIC Homodimerization. Chem. Biol. 22 (7), 838–848. 10.1016/j.chembiol.2015.06.008 26144885

[B3] Aziz T. A. Hussain S. A. Mahwi T. O. Ahmed Z. A. Rahman H. S. Rasedee A. (2018). The Efficacy and Safety of Ginkgo Biloba Extract as an Adjuvant in Type 2 Diabetes Mellitus Patients Ineffectively Managed with Metformin: a Double-Blind, Randomized, Placebo-Controlled Trial. Drug Des. Devel Ther. 12, 735–742. 10.2147/DDDT.S157113 PMC5896648 29670330

[B4] Bai H. Zhang Z. Li Y. Song X. Ma T. Liu C. (2020). L-theanine Reduced the Development of Knee Osteoarthritis in Rats via its Anti-inflammation and Anti-matrix Degradation Actions: *In Vivo* and *In Vitro* Study. Nutrients 12 (7). 10.3390/nu12071988 PMC7400703 32635404

[B5] Benjamin J. Muir T. Briggs K. Pentland B. (2001). A Case of Cerebral Haemorrhage-Can Ginkgo Biloba Be Implicated? Postgrad. Med. J. 77 (904), 112–113. 10.1136/pmj.77.904.112 11161079 PMC1741913

[B6] Chen Y. F. Jobanputra P. Barton P. Bryan S. Fry-Smith A. Harris G. (2008). Cyclooxygenase-2 Selective Non-steroidal Anti-inflammatory Drugs (Etodolac, Meloxicam, Celecoxib, Rofecoxib, Etoricoxib, Valdecoxib and Lumiracoxib) for Osteoarthritis and Rheumatoid Arthritis: a Systematic Review and Economic Evaluation. Health Technol. Assess. 12 (11), 1–iii. 10.3310/hta12110 18405470

[B7] Cooper C. Chapurlat R. Al-Daghri N. Herrero-Beaumont G. Bruyère O. Rannou F. (2019). Safety of Oral Non-selective Non-steroidal Anti-inflammatory Drugs in Osteoarthritis: What Does the Literature Say? Drugs Aging 36 (Suppl. 1), 15–24. 10.1007/s40266-019-00660-1 31073921 PMC6509083

[B8] dal Belo S. E. Gaspar L. R. Maia Campos P. M. Marty J. P. (2009). Skin Penetration of Epigallocatechin-3-Gallate and Quercetin from green tea and Ginkgo Biloba Extracts Vehiculated in Cosmetic Formulations. Skin Pharmacol. Physiol. 22 (6), 299–304. 10.1159/000241299 19786823

[B9] De Smet P. A. (2002). Herbal Remedies. N. Engl. J. Med. 347 (25), 2046–2056. 10.1056/NEJMra020398 12490687

[B10] Duan R. Xie H. Liu Z. Z. (2020). The Role of Autophagy in Osteoarthritis. Front Cell Dev Biol 8, 608388. 10.3389/fcell.2020.608388 33324654 PMC7723985

[B11] Feng Z. Sun Q. Chen W. Bai Y. Hu D. Xie X. (2019). The Neuroprotective Mechanisms of Ginkgolides and Bilobalide in Cerebral Ischemic Injury: a Literature Review. Mol. Med. 25 (1), 57. 10.1186/s10020-019-0125-y 31864312 PMC6925848

[B12] Ge Y. Huang M. Yao Y. M. (2018). Autophagy and Proinflammatory Cytokines: Interactions and Clinical Implications. Cytokine Growth Factor. Rev. 43, 38–46. 10.1016/j.cytogfr.2018.07.001 30031632

[B13] Goldie M. Dolan S. (2013). Bilobalide, a Unique Constituent of Ginkgo Biloba, Inhibits Inflammatory Pain in Rats. Behav. Pharmacol. 24 (4), 298–306. 10.1097/FBP.0b013e32836360ab 23838965

[B14] Grässel S. Zaucke F. Madry H. (2021). Osteoarthritis: Novel Molecular Mechanisms Increase Our Understanding of the Disease Pathology. J. Clin. Med. 10 (9). 10.3390/jcm10091938 PMC8125020 33946429

[B15] Heinrich M. Appendino G. Efferth T. Fürst R. Izzo A. A. Kayser O. (2020). Best Practice in Research - Overcoming Common Challenges in Phytopharmacological Research. J. Ethnopharmacol 246, 112230. 10.1016/j.jep.2019.112230 31526860

[B16] Henrotin Y. Mobasheri A. (2018). Natural Products for Promoting Joint Health and Managing Osteoarthritis. Curr. Rheumatol. Rep. 20 (11), 72. 10.1007/s11926-018-0782-9 30232562

[B17] Holczer M. Hajdú B. Lőrincz T. Szarka A. Bánhegyi G. Kapuy O. (2019). A Double Negative Feedback Loop between mTORC1 and AMPK Kinases Guarantees Precise Autophagy Induction upon Cellular Stress. Int. J. Mol. Sci. 20 (22). 10.3390/ijms20225543 PMC6888297 31703252

[B18] Ilieva I. Ohgami K. Shiratori K. Koyama Y. Yoshida K. Kase S. (2004). The Effects of Ginkgo Biloba Extract on Lipopolysaccharide-Induced Inflammation *In Vitro* and *In Vivo* . Exp. Eye Res. 79 (2), 181–187. 10.1016/j.exer.2004.03.009 15325565

[B19] Im H. J. Kim J. S. Li X. Kotwal N. Sumner D. R. van Wijnen A. J. (2010). Alteration of Sensory Neurons and Spinal Response to an Experimental Osteoarthritis Pain Model. Arthritis Rheum. 62 (10), 2995–3005. 10.1002/art.27608 20556813 PMC2952041

[B20] Jenei-Lanzl Z. Meurer A. Zaucke F. (2019). Interleukin-1β Signaling in Osteoarthritis - Chondrocytes in Focus. Cell Signal 53, 212–223. 10.1016/j.cellsig.2018.10.005 30312659

[B21] Jiang M. Li J. Peng Q. Liu Y. Liu W. Luo C. (2014). Neuroprotective Effects of Bilobalide on Cerebral Ischemia and Reperfusion Injury Are Associated with Inhibition of Pro-inflammatory Mediator Production and Down-Regulation of JNK1/2 and P38 MAPK Activation. J. Neuroinflammation 11, 167. 10.1186/s12974-014-0167-6 25256700 PMC4189683

[B22] Jiang W. Liu H. Wan R. Wu Y. Shi Z. Huang W. (2021). Mechanisms Linking Mitochondrial Mechanotransduction and Chondrocyte Biology in the Pathogenesis of Osteoarthritis. Ageing Res. Rev. 67, 101315. 10.1016/j.arr.2021.101315 33684550

[B23] Kang C. Elledge S. J. (2016). How Autophagy Both Activates and Inhibits Cellular Senescence. Autophagy 12 (5), 898–899. 10.1080/15548627.2015.1121361 27129029 PMC4854549

[B24] Katri A. Dąbrowska A. Löfvall H. Karsdal M. A. Andreassen K. V. Thudium C. S. (2019). A Dual Amylin and Calcitonin Receptor Agonist Inhibits Pain Behavior and Reduces Cartilage Pathology in an Osteoarthritis Rat Model. Osteoarthritis Cartilage 27 (9), 1339–1346. 10.1016/j.joca.2019.05.016 31176015

[B25] Katz J. N. Arant K. R. Loeser R. F. (2021). Diagnosis and Treatment of Hip and Knee Osteoarthritis: A Review. JAMA 325 (6), 568–578. 10.1001/jama.2020.22171 33560326 PMC8225295

[B26] Lee I. H. Cao L. Mostoslavsky R. Lombard D. B. Liu J. Bruns N. E. (2008). A Role for the NAD-dependent Deacetylase Sirt1 in the Regulation of Autophagy. Proc. Natl. Acad. Sci. U S A. 105 (9), 3374–3379. 10.1073/pnas.0712145105 18296641 PMC2265142

[B27] Ma T. w. Wen Y. j. Song X. p. Hu H. l. Li Y. Bai H. (2020). Puerarin Inhibits the Development of Osteoarthritis through Antiinflammatory and Antimatrix‐degrading Pathways in Osteoarthritis‐induced Rat Model. Phytotherapy Res. 35, 2579–2593. 10.1002/ptr.6988 33350519

[B28] Ma Y. Liu H. Du X. Petlulu P. Chen X. Wang R. (2021). IRE1 and CaMKKβ Pathways to Reveal the Mechanism Involved in Microcystin-LR-Induced Autophagy in Mouse Ovarian Cells. Food Chem. Toxicol. 147, 111911. 10.1016/j.fct.2020.111911 33290805

[B29] Mahadevan S. Park Y. (2008). Multifaceted Therapeutic Benefits of Ginkgo Biloba L.: Chemistry, Efficacy, Safety, and Uses. J. Food Sci. 73 (1), R14–R19. 10.1111/j.1750-3841.2007.00597.x 18211362

[B30] Mao D. Li H. Zhang L. Xu J. Yu C. Zhang Q. (2019). Bilobalide Alleviates IL-17-induced Inflammatory Injury in ATDC5 Cells by Downregulation of microRNA-125a. J. Biochem. Mol. Toxicol. 33 (12), e22405. 10.1002/jbt.22405 31593333

[B31] Mei R. Lou P. You G. Jiang T. Yu X. Guo L. (2020). 17β-Estradiol Induces Mitophagy Upregulation to Protect Chondrocytes via the SIRT1-Mediated AMPK/mTOR Signaling Pathway. Front. Endocrinol. 11, 615250. 10.3389/fendo.2020.615250 PMC7888342 33613450

[B32] Netea-Maier R. T. Plantinga T. S. van de Veerdonk F. L. Smit J. W. Netea M. G. (2016). Modulation of Inflammation by Autophagy: Consequences for Human Disease. Autophagy 12 (2), 245–260. 10.1080/15548627.2015.1071759 26222012 PMC4836004

[B33] Palacios S. Soler E. Ramírez M. Lilue M. Khorsandi D. Losa F. (2019). Effect of a Multi-Ingredient Based Food Supplement on Sexual Function in Women with Low Sexual Desire. BMC Womens Health 19 (1), 58. 10.1186/s12905-019-0755-9 31039769 PMC6492381

[B34] Panahifar A. Jaremko J. L. Tessier A. G. Lambert R. G. Maksymowych W. P. Fallone B. G. (2014). Development and Reliability of a Multi-Modality Scoring System for Evaluation of Disease Progression in Pre-clinical Models of Osteoarthritis: Celecoxib May Possess Disease-Modifying Properties. Osteoarthritis Cartilage 22 (10), 1639–1650. 10.1016/j.joca.2014.06.013 25278073

[B35] Pei W. Huang X. Ni B. Zhang R. Niu G. You H. (2021). Selective STAT3 Inhibitor Alantolactone Ameliorates Osteoarthritis via Regulating Chondrocyte Autophagy and Cartilage Homeostasis. Front. Pharmacol. 12, 730312. 10.3389/fphar.2021.730312 34650433 PMC8505527

[B36] Pérez-Lozano M. L. Cesaro A. Mazor M. Esteve E. Berteina-Raboin S. Best T. M. (2021). Emerging Natural-Product-Based Treatments for the Management of Osteoarthritis. Antioxidants (Basel) 10 (2). 10.3390/antiox10020265 PMC7914872 33572126

[B37] Piel M. J. Kroin J. S. van Wijnen A. J. Kc R. Im H. J. (2014). Pain Assessment in Animal Models of Osteoarthritis. Gene 537 (2), 184–188. 10.1016/j.gene.2013.11.091 24333346 PMC3950312

[B38] Pritzker K. P. Gay S. Jimenez S. A. Ostergaard K. Pelletier J. P. Revell P. A. (2006). Osteoarthritis Cartilage Histopathology: Grading and Staging. Osteoarthritis Cartilage 14 (1), 13–29. 10.1016/j.joca.2005.07.014 16242352

[B39] Qin Y. R. Ma C. Q. Wang D. P. Zhang Q. Q. Liu M. R. Zhao H. R. (2021). Bilobalide Alleviates Neuroinflammation and Promotes Autophagy in Alzheimer's Disease by Upregulating lincRNA-P21. Am. J. Transl Res. 13 (4), 2021–2040. 34017373 PMC8129331

[B40] Rong X. Xu J. Jiang Y. Li F. Chen Y. Dou Q. P. (2021). Citrus Peel Flavonoid Nobiletin Alleviates Lipopolysaccharide-Induced Inflammation by Activating IL-6/STAT3/FOXO3a-mediated Autophagy. Food Funct. 12 (3), 1305–1317. 10.1039/d0fo02141e 33439200

[B41] Sawa-Makarska J. Baumann V. Coudevylle N. von Bülow S. Nogellova V. Abert C. (2020). Reconstitution of Autophagosome Nucleation Defines Atg9 Vesicles as Seeds for Membrane Formation. Science 369 (6508). 10.1126/science.aaz7714 PMC7610778 32883836

[B42] Sheu S. Y. Ho S. R. Sun J. S. Chen C. Y. Ke C. J. (2015). Arthropod Steroid Hormone (20-Hydroxyecdysone) Suppresses IL-1β-induced Catabolic Gene Expression in Cartilage. BMC Complement. Altern. Med. 15, 1. 10.1186/s12906-015-0520-z 25617057 PMC4310028

[B43] Sukhikh S. Noskova S. Ivanova S. Ulrikh E. Izgaryshev A. Babich O. (2021). Chondroprotection and Molecular Mechanism of Action of Phytonutraceuticals on Osteoarthritis. Molecules 26 (8), 2391. 10.3390/molecules26082391 33924083 PMC8074261

[B44] Tanida I. (2011). Autophagosome Formation and Molecular Mechanism of Autophagy. Antioxid. Redox Signal. 14 (11), 2201–2214. 10.1089/ars.2010.3482 20712405

[B45] Tian Z. Zhang X. Sun M. (2021). Phytochemicals Mediate Autophagy against Osteoarthritis by Maintaining Cartilage Homeostasis. Front. Pharmacol. 12, 795058. 10.3389/fphar.2021.795058 34987406 PMC8722717

[B46] Tsai S. W. Lin C. C. Lin S. C. Wang S. P. Yang D. H. (2019). Isorhamnetin Ameliorates Inflammatory Responses and Articular Cartilage Damage in the Rats of Monosodium Iodoacetate-Induced Osteoarthritis. Immunopharmacol Immunotoxicol 41 (4), 504–512. 10.1080/08923973.2019.1641723 31342791

[B47] Tu C. Huang X. Xiao Y. Song M. Ma Y. Yan J. (2019). Schisandrin A Inhibits the IL-1β-Induced Inflammation and Cartilage Degradation via Suppression of MAPK and NF-Κb Signal Pathways in Rat Chondrocytes. Front. Pharmacol. 10, 41. 10.3389/fphar.2019.00041 30761007 PMC6361757

[B48] Vale S. (1998). Subarachnoid Haemorrhage Associated with Ginkgo Biloba. Lancet 352 (9121), 36. 10.1016/S0140-6736(05)79516-7 9800751

[B49] Valenti M. T. Dalle Carbonare L. Zipeto D. Mottes M. (2021). Control of the Autophagy Pathway in Osteoarthritis: Key Regulators, Therapeutic Targets and Therapeutic Strategies. Ijms 22 (5), 2700. 10.3390/ijms22052700 33800062 PMC7962119

[B50] Vinatier C. Domínguez E. Guicheux J. Caramés B. (2018). Role of the Inflammation-Autophagy-Senescence Integrative Network in Osteoarthritis. Front. Physiol. 9, 706. 10.3389/fphys.2018.00706 29988615 PMC6026810

[B51] Wang C. Yao Z. Zhang Y. Yang Y. Liu J. Shi Y. (2020). Metformin Mitigates Cartilage Degradation by Activating AMPK/SIRT1-Mediated Autophagy in a Mouse Osteoarthritis Model. Front. Pharmacol. 11, 1114. 10.3389/fphar.2020.01114 32792951 PMC7393141

[B52] Wang F. Liu J. Chen X. Zheng X. Qu N. Zhang B. (2019). IL-1β Receptor Antagonist (IL-1Ra) Combined with Autophagy Inducer (TAT-Beclin1) Is an Effective Alternative for Attenuating Extracellular Matrix Degradation in Rat and Human Osteoarthritis Chondrocytes. Arthritis Res. Ther. 21 (1), 171. 10.1186/s13075-019-1952-5 31291980 PMC6617669

[B53] Wang T. He C. (2018). Pro-inflammatory Cytokines: The Link between Obesity and Osteoarthritis. Cytokine Growth Factor. Rev. 44, 38–50. 10.1016/j.cytogfr.2018.10.002 30340925

[B54] Yan J. Ni B. Sheng G. Zhang Y. Xiao Y. Ma Y. (2021). Rhoifolin Ameliorates Osteoarthritis via Regulating Autophagy. Front. Pharmacol. 12, 661072. 10.3389/fphar.2021.661072 34122080 PMC8194266

[B55] Yang Z. Klionsky D. J. (2010). Mammalian Autophagy: Core Molecular Machinery and Signaling Regulation. Curr. Opin. Cell Biol 22 (2), 124–131. 10.1016/j.ceb.2009.11.014 20034776 PMC2854249

[B56] Ye J. Ye C. Huang Y. Zhang N. Zhang X. Xiao M. (2019). Ginkgo Biloba Sarcotesta Polysaccharide Inhibits Inflammatory Responses through Suppressing Both NF-Κb and MAPK Signaling Pathway. J. Sci. Food Agric. 99 (5), 2329–2339. 10.1002/jsfa.9431 30338529

[B57] Zhang H. Cao N. Yang Z. Fang X. Yang X. Li H. (2020a). Bilobalide Alleviated Dextran Sulfate Sodium-Induced Experimental Colitis by Inhibiting M1 Macrophage Polarization through the NF-Κb Signaling Pathway. Front. Pharmacol. 11, 718. 10.3389/fphar.2020.00718 32670051 PMC7326085

[B58] Zhang M. Kenny S. J. Ge L. Xu K. Schekman R. (2015). Translocation of Interleukin-1β into a Vesicle Intermediate in Autophagy-Mediated Secretion. Elife 4. 10.7554/eLife.11205 PMC4728131 26523392

[B59] Zhang M. Liu Y. Huan Z. Wang Y. Xu J. (2020b). Metformin Protects Chondrocytes against IL-1β Induced Injury by Regulation of the AMPK/NF-κ B Signaling Pathway. Pharmazie 75 (12), 632–636. 10.1691/ph.2020.0762 33303055

[B60] Zhao M. Qin J. Shen W. Wu A. (2021). Bilobalide Enhances AMPK Activity to Improve Liver Injury and Metabolic Disorders in STZ-Induced Diabetes in Immature Rats via Regulating HMGB1/TLR4/NF-Κb Signaling Pathway. Biomed. Res. Int. 2021, 8835408. 10.1155/2021/8835408 33959665 PMC8075671

[B61] Zheng L. Zhang Z. Sheng P. Mobasheri A. (2021). The Role of Metabolism in Chondrocyte Dysfunction and the Progression of Osteoarthritis. Ageing Res. Rev. 66, 101249. 10.1016/j.arr.2020.101249 33383189

[B62] Zheng Y. Wu Z. Yi F. Orange M. Yao M. Yang B. (2018). By Activating Akt/eNOS Bilobalide B Inhibits Autophagy and Promotes Angiogenesis Following Focal Cerebral Ischemia Reperfusion. Cell Physiol Biochem 47 (2), 604–616. 10.1159/000490016 29794436

[B63] Zhou H. Li G. Wang Y. Jiang R. Li Y. Wang H. (2021). Microbial Metabolite Sodium Butyrate Attenuates Cartilage Degradation by Restoring Impaired Autophagy and Autophagic Flux in Osteoarthritis Development. Front. Pharmacol. 12, 659597. 10.3389/fphar.2021.659597 33897442 PMC8062861

